# Achieving ‘something that everybody has invested in’: perspectives of diverse stakeholders during co-creation of a transition to residency curriculum

**DOI:** 10.1186/s12909-024-05573-1

**Published:** 2024-06-11

**Authors:** Shireen Suliman, Margaret Allen, Ayad Al-Moslih, Alison Carr, Richard Koopmans, Karen D. Könings

**Affiliations:** 1 Medical Education, Hamad Medical Corporation, Doha, Qatar; 2 College of Medicine, QU Health, Qatar University, Doha, Qatar; 3 Medicine, Weill Cornell Medicine Qatar, Doha, Qatar; 4 School of Health Professions Education, Maastricht University, Maastricht, The Netherlands; 5 Medical Education Department, Hamad Medical Corporation, Doha, Qatar; 6 Disaster Medicine, University of Central Lancashire, Preston, England; 7 School of Medicine and Dentistry, University of Central Lancashire, Preston, England; 8 Internal Medicine, Maastricht University Medical Center+, Maastricht, The Netherlands; 9 School of Health Sciences, University of East Anglia, Norwich, UK

**Keywords:** Co-creation, Diverse stakeholders, Transition to residency, Undergraduate medical education, Graduate medical education

## Abstract

Co-creation of education within the context of student inclusion alongside *diverse* stakeholders merits exploration. We studied the perspectives of students and teachers from different institutions who participated in co-creating a transition to residency curriculum. We conducted post-hoc in-depth interviews with 16 participants: final-year medical students, undergraduate, and postgraduate medical education stakeholders who were involved in the co-creation sessions. Findings build on the Framework of Stakeholders’ Involvement in Co-creation and identify the four key components of co-creation with diverse faculty: immersion in positive feelings of inclusivity and appreciation, exchange of knowledge, engagement in a state of reflection and analysis, and translation of co-creation dialogues into intended outcomes. Despite power dynamics, participants valued open communication, constructive feedback, mutual respect, and effective moderation. The study broadened our understanding of the co-creation process in diverse stakeholder settings. Incorporating key elements in the presence of power relations can enrich co-creation by leveraging wider expertise.

**Supplementary Information:**

The online version contains supplementary material available at 10.1186/s12909-024-05573-1.

## Introduction

Co-creation (CC) has been valued as a promising strategy for teacher-learner collaboration in designing education that enhances the sense of learner belonging, empowerment, motivation, and confidence and the quality of educational design [1–3]. The Framework of Stakeholders’ Involvement in Co-creation (Könings et al., 2021) depicts the importance of active involvement of not just learners and teachers, but also other relevant stakeholders such as workplace partners, nurses, patients, or educational designers [4, 5]. CC can improve both student learning and teacher practices, while also benefiting a wider population with more context-appropriate educational designs [6]. With potential problematic dynamics, essentially the power relations between learners and teachers [3, 6, 7], there is a need to explore the multi-stakeholder CC-process, including students together with diverse faculty.

Medical graduates move from undergraduate education (UME) to graduate education (GME). Medical schools prepare medical students for postgraduate training [8], but GME stakeholders also benefit from having a voice in student preparation before they engage with their organizations [9]. They can evaluate students’ preparedness, determine graduates’ skills, and make recommendations [10]. Yet, suboptimal communication between medical schools and residency programs contributes to a ‘transition gap’ [11]. Efforts to bridge this gap involve sharing information between faculty supervisors [12]. However, poor coordination across the educational continuum creates stress for all stakeholders [13] with a lack of shared responsibility for necessary curricular modifications [14]. With the introduction of CC for improving the education program during the transition to residency [15], it is opportune to explore stakeholders’ perspectives and group dynamics in the context of multi-stakeholder CC and investigate whether CC participates in convening collaboration between responsible UME and GME stakeholders.

Stakeholders’ presence in the CC-process does not guarantee successful collaboration. The Positioning Theory proposes that individuals adopt specific positions that come with certain rights, obligations, and duties for appropriate ways of communication and conduct during interactions. In a particular social context, a set of rights and duties accepted by a group of people is referred to as a position. The act of positioning is the corresponding action through which someone claims particular rights, chooses specific duties, or has them imposed upon them by a particular social actor [16]. It includes three core analytical concepts: (1) position, a set of rights and obligations that dictate actions; (2) action, including speech acts; (3) storylines, which denote the episode’s moral and social order [17, 18]. Therefore, despite the need for diversity to reveal different perspectives and ideas [19], diverse stakeholders might play isolated roles that could limit their experience of ownership in the overall process and outputs, risking the group dynamics [9]. University staff face the challenge of relinquishing power when co-creating with students [20]. UME and GME faculty have differing perspectives on graduates’ preparedness for residency [21] and involving diverse faculty may hinder the CC-process with students. As the theory can guide the understanding of social practices in learner-teacher interactions [22], it can provide a lens to interpret the dynamics of CC involving diverse faculty. Limited research has explored including workplace partners in CC to redistribute power between students and faculty [9]. We aim to examine the dynamics of CC involving diverse faculty and students that allow successful collaboration.

We recently set up a CC initiative where students, together with UME and GME responsible stakeholders were involved in co-creating a ‘transition to residency’ curriculum, fully described by Suliman, Könings [15]. This study, entitled by a participant quote, explores how participants experience the CC-process of students with diverse faculty across the transition spectrum. We explored participants’ perceptions of factors facilitating the CC process as well as the impact of power dynamics withing the group, and ways to mitigate them.

## Materials and methods

We used a constructivist grounded theory approach and qualitative methodology and conducted post-hoc in-depth interviews [23] with 16 participants among those involved in the co-creation sessions (CCSs) of the transition to residency curriculum between January and March 2021.

### Synopsis of the CCSs

Using purposeful sampling, 46 participants were recruited from two institutions: Qatar University College of Medicine and Hamad Medical Corporation, Qatar’s major GME training organization, which holds Accreditation Council for Graduate Medical Education International accreditation. These were representative of three key stakeholder groups across the transition curriculum: 23 final-year medical students, nine UME stakeholders (leaders and faculty involved in assessment and curriculum committees), and 14 GME stakeholders such as residency program directors, core faculty, and chief residents. A total of 10 online CCSs were conducted resulting in a Model of Learning during Transition [15] containing five elements: Adaptation, authenticity, autonomy, connectedness, and continuity; all were embedded in the foundation of a supportive environment.

### Interview participants and sampling

As purposive sampling was used to recruit 46 CCS participants from the three stakeholder groups, the convenience sampling technique was the approach for the interview participants, as all were information-rich [24]. All participants received email invitations with attached informed consent, signed and returned by participants via email before the interviews. The invitation included a thorough description of the study purpose with emphasis on voluntary participation and confidentiality. The respondents included GME stakeholders (*n* = 5), UGE stakeholders (*n* = 3), and final-year medical students (*n* = 8; 5 women, 3 men). Six faculty were men and two were women. The five chief residents were invited but did not respond. After 16 interviews, the research team gained an adequate understanding and thus claimed data saturation for the recruited stakeholder groups [25].

### Data collection

Following the CCSs, we conducted hour-long semi-structured interviews using two guides (Supplementary Appendices A and B) which started with a literature-based definition of CC: “when staff and students work collaboratively with one another to create components of curricula and/or pedagogical approaches” [3]. All interviews began with an open question such as “Describe your experience with CC”, and the subsequent answers were then explored with more specific questions. Questions for students included: “Did you feel comfortable contributing?“, “Did you feel valued and listened to?“, “How did your contribution add value to the curriculum?“. Faculty were asked to describe their experience with CC and their thoughts on student input. All interviews were recorded and transcribed. All participants verified and agreed on the one-page summary of their interview transcript (member check). All responses were pseudonymized by coding participants’ names and de-identifying all quotes.

### Data analysis

Following a grounded theory methodology, we conducted an iterative approach and used emergent themes to refine the interview guide [23]. Authors (SS and AA) performed open coding for the first two interviews independently, condensing them to categories using constant comparative analysis of emerging characteristics and properties of the category, among different perceptions and readings of the data [26, 27]. Then, authors SS and AA discussed and agreed upon other categories and developed a coding scheme. MA, AC, and KK then adjusted the coding scheme, and the entire research team revised and approved it. The main author (SS) then analysed all remaining interviews using (but not restricted to) the coding scheme and returned to previous transcripts when new codes emerged. We used Atlas. ti qualitative software, version 9.4.0 for coding and data management.

The study received expedited ethical approval by Qatar University Institutional Review Board (QU-IRB1348-EA/20) and Hamad Medical Corporation (HMC)- Medical Research Centre (MRC) Ethical Review Board (HMC- MRC-01-20-265).

### Reflexivity

All authors have a background in medical education and paid great attention to their practices and judgment during data collection and analysis, to mitigate bias or influence. To ensure fairness and avoid power imbalances, authors interviewed stakeholders outside their institution, promoting openness. SS engaged in reflexivity by writing memos during data collection and analysis and met frequently with the research team during coding and revision of the coding scheme. Co-investigators KK, MA, and RK were experienced researchers who provided supervision and ensured ethical conduct during the study.

## Results

Two main themes emerged in our analysis. The first theme constitutes four core components of the CC-process in the presence of diverse faculty: the immersion in positive feelings of inclusivity and appreciation, exchange of knowledge, engagement in a state of reflection and analysis, and translation of CC dialogues into intended outcomes. The second theme describes the dual power relations in the presence of diverse faculty: the student-faculty and faculty-faculty power relations, and their mitigating factors. We will describe the themes/subthemes using supporting quotes where UME stakeholders are identified with (U), GME stakeholders (G), and medical students with (S).

### Core components of the multi-stakeholder CC-process

The following section describes the four core components that participants described during the CC in the presence of diverse faculty and students.

### Immersion in positive feelings of inclusivity and appreciation

Feeling happy about “*being involved and hearing ideas from each other” (G1)*, the GME stakeholders valued students’ input and thought *“highly of them” (G1).* They emphasized that it is important to include students in planning transition curricula as they *“are the ones who are facing the students when they are coming” (G2).* Similarly, UGE stakeholders had a positive perception of CC as it made them feel “*happy to hear ideas and things from my colleagues and the students” (U1).* They appreciated students’ input as they *“can teach so much about how to do the transition in a better way that we can prepare the students for it and they can survive it in a good way” (U1).* Students also felt happy and *“good to be involved” (S1)* and also empowered as they *“liked having the choice to participate in curriculum adjustment” (S2).* Students found faculty *“very appreciating” (S3); they “welcomed”* and *“valued their opinions”(S4)* and “*are taking it into consideration and putting it in the curriculum”(S5).* Moreover, students expressed a feeling of reduced stress by saying: *“Having an expectation of what we are doing next year is a nice feeling, like, you get to feel relaxed and less anxious” (S6). Another student added “Involving the students, maybe relieves some of the stress because I feel, if the college does the entire work on the curriculum, this might like, create unreasonable or very high expectations on the students, and this will create high stress. But if the students are involved and they would tell the college if this is too much, or if it’s too little, then there’s the middle ground where the expectations are not too high but not too low and both know what a reasonable expectation would be”(S4).*

### Exchange of knowledge

Besides these positive feelings, students valued the inclusion of diverse faculty *“because both of them have different perspectives”(S6)*, clarified “*the objectives to be achieved”(S3)* in the preclinical years and at the same time *“what the program directors are expecting”(S6)* from them when they join as residents. It also allowed them to state their needs because *“students points would be the most precious points because if you take the feedback from the students, then absolutely, you’ll improve for the next students or for the next batch”(S7)* and “*even though teachers or faculty are experienced with developing curriculum, they won’t see, the perspective that the students see or feel what is important for the students”(S6).* They informed the *“clinicians about what do they think of the curriculum”(S2)* and provided suggestions for improvement so faculty will not *“miss some of the important skills that are needed for a graduated student”(S7)* such as courses in Ethics. Faculty were informed and came to *“know what we have taken and what we have not covered”(S3).*

Describing it as a *“learning opportunity”(G5)*, GME stakeholders found CC an opportunity to connect with the students and be informed about their preparation and needs. They came *“to know what they are learning”(G3) and “where they are and what can be developed more”(G5).* They were also informed by their UGE colleagues who*“have brought a different angle because they are real faculty within the college, they did give the students all the time and they know exactly what the students want”(G2).* The UME appreciated their GME colleagues and labeled them as *“the most valuable resources of the college”(U2)* because *“they have the rich experience in the field”(U1)* and *“know more about what they really need and what they really like so they can help them”(U1).* Similar to GME stakeholders, they described CC as *“a learning opportunity, and a kind of handover”(U3)* that allows them to inform their GME fellows about students’ preparation as they “*might be missing things that we are aware of” (U2)* and at the same time be informed about residency programs expectations and *“and what needs to be done”(U3)* to prepare students. They felt that listening to students “*is a good way to make sure that we’re hearing their voices and understanding what they’re worried about and what they want to address”(U3).*

### Engagement in a state of reflection and analysing

Having been informed about GME expectations, students engaged in reflective experience during CC stating: “*We explored our weaknesses and also our gaps in knowledge and skills …. and then also to see in which area we are stronger … and this will help us, of course, in our future career because now we know what we know, and we don’t know”(S3).* This allowed them to prioritize learning for example: “*it’s important to know how to diagnose patients first, and then to think about management later”(S5).* Likewise, UME stakeholders perceived CC as means of “*more of self-evaluation and mirroring to myself about my knowledge, and this is the added value of it, this was the enjoyable part of it”(U1).* The involvement of the students in CC made UME stakeholders think *“about the things in a different way and how to incorporate things differently in the curriculum”(U1)* and *“make necessary changes that will be student-oriented and friendly”(U2)* so that students *“have more enjoyable rich experience”(U1).* UME stakeholders believed that their contribution to CC helped students as well in integrating “*the theoretical parts within the practical context”(U2).* GME stakeholders reflected on the curriculum as well looking at what “*is missing in that one or what going to be done better”(G2)*. They added that: *“it is very important to have a base of understanding at the beginning and then visit and revisit the curriculum to iron out the missed problems and that should have an impact on the quality of the training”(G2).*

### Translation of CC dialogues into intended outcomes

Participants emphasized the importance of following up on the CC dialogues and making *“changes based on these opinions”(U2).* Students believed that *“co-creation is meant to take the ideas from the students and implement them in the curriculum, and so, if you implement what we feel is missing or what we feel we need more practice on, of course, will help with the training overall”(S6)*. Likewise, GME stakeholders thought that the information generated through CC, *“might be useful for the curriculum and add value to the curriculum”(G4).* Finally, participants believed that convening the UGE and GME stakeholders with students during CC resulted in *“something that everybody invested in, everybody knows about and everybody thinks works”(G3).*

### The dual power relations in the presence of diverse faculty

#### Student-faculty power relations

Students expressed some discomfort in voicing their opinions in the presence of both UME and GME stakeholders. Students expressed that *“there was a little bit of a holding back in having a co-creation with someone expert in curricula” (S6)* and in the presence of *“someone who might be program director in the future”(S6)* or responsible for students’ evaluation. Moreover, students feared being *“disliked”(S3)* by their UME stakeholders whom they are familiar with. Yet while students “*felt afraid to speak freely”(S8)*, this mainly/solely happened *“at the beginning”(S8)* of the session. Later *“everything went so like, professionally”(S6)* and their voices were listened to and their *“input was fully respected”(S3).* While students perceive these power dynamics, faculty appreciated that students were *“much more engaged with the process”(U3)* and *“spoke up”(G1)*, with a *“climate of confidence”(G4)* and *“mass contribution”(G2)* and *“actively involved”(U2)* in discussions. Thus, power dynamics between students and the diverse faculty were perceived by students as a temporary discomfort, while positive feelings dominated in faculty.

#### Faculty-faculty power relations

When asked about CC-experiences with their fellow GME, the UME stakeholders felt *“very comfortable being with them”(U3)*. Despite differences in opinions, one of the UME stakeholders stated: *“I understood his perspective, of course, my perspective was a bit different, but then it’s okay”(U3).* They added that they did not feel that *“the clinicians do not want to hear or have kind of rejection to listen … they were willing to listen and even if they, might have a different idea, they did not let’s say, reveal or express rejection or any superiority”(U1).* Similarly, GME stakeholders valued UME colleagues as they were “*able to give their contribution and what they wanted and what they expected, and it is an addition it is a valuable addition”(G2).*

#### Factors mitigating the power relations

In the presence of diverse faculty, participants appreciated a healthy discussion environment, which they attributed to several factors. First, CC was labeled as an “*open discussion between different parties”(G2)* where everyone felt free to participate and express their thoughts. Second, discussions were characterized by *“constructive feedback rather than criticism”(U3)* which nurtured a safe environment for discussion. Third, there was a perception of mutual respect where students stated that *“our opinions and our input were fully respected” (S3)* and they *“were discussing each other’s comments with respect, and everyone showed respect”(S6).* Equally, faculty perceived*“no hierarchy of knowledge….and reflections and contributions were treated equally”(U1)*, and students showed respect “*when they have provided their opinion”(G4).* Finally, the role of the moderator during CC was critical. Described by participants as an *“important player”(U1)*, the moderator *“guided a respectful discussion”(U1)* and “*allowed everybody to contribu1te”(S4)* and emphasized that *“everybody has something to contribute so you empower them, you (the moderator) validate them and you get the best information from them”(G3).* They described certain desirable characteristics to be present in the session moderator which include: being wise, calm, appreciative, neutral *“don’t have a relation with those students”(S3)*, and non-judgemental. They created a healthy environment by having a structured guide of questions, inviting all participants to contribute, controlling the discussion, and *“calm the things and prevent tense that can result from sentence or word that anyone says”(U1).* They made participants feel*“comfortable because the way the questions we have were generalized, not individualized, so we were listening to each other” (G5).*

## Discussion

**Fig. 1:**
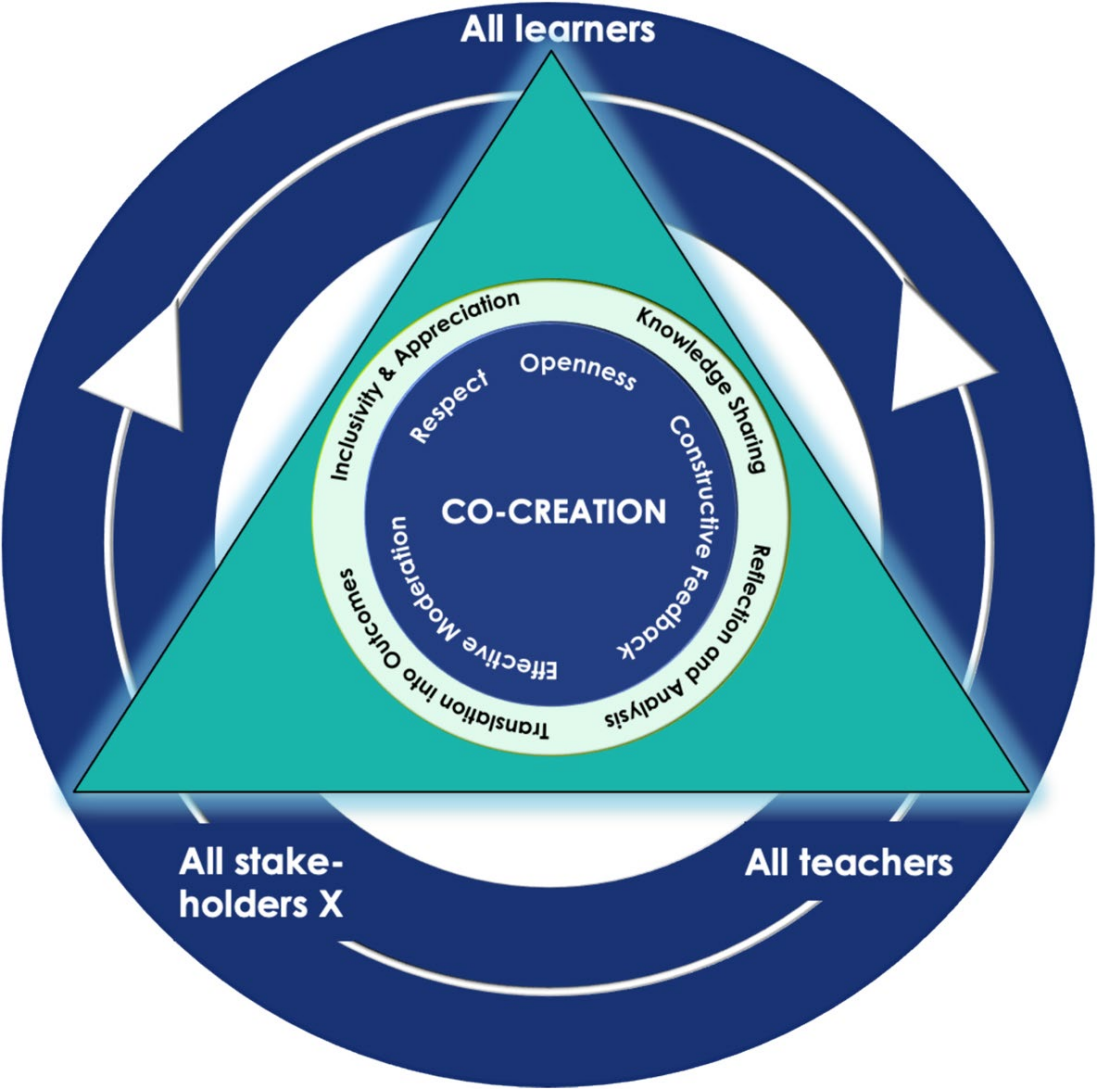
Modified Framework of Stakeholder Involvement in Co-Creation, Visualizing the Findings of the Current Study. Note. The triangle denotes the co-creation participants, in this study: Learners – final year medical students; Teachers – UME stakeholders; and Stakeholders X - GME stakeholders. Displayed within the triangle in the middle are the four core components of the CC-process that involve diverse faculty: Inclusivity and appreciation, knowledge sharing, reflection and analysis of current practices, and translation of co-creation dialogues into outcomes. The mitigating factors of power relations: Openness, respect, constructive feedback, and effective moderation are engrained in the core of the figure as the central fundamentals for the CC-process

 This study aimed to enhance our understanding of the CC-process that involves students with diverse faculty. Previous work mainly focused on the CC of students with university staff. The unique perspective of this study arises from the inclusion of students together with faculty from different institutions that sit at the extreme ends of transition. We incorporated the emerging themes in the core of the Stakeholders’ Involvement Framework for Co-creation, originally described by Könings, Mordang [6] (Fig. 1). The triangle in the figure denotes the CC participants: the learners being final year medical students, the workplace partners being GME stakeholders, and the teachers being UGE stakeholders. As stated by the Positioning Theory, the UME and GME stakeholder interactions with students are expected to be guided by the positions they assume during CC, resulting in positions-driven dual student-faculty and faculty-faculty power relations. Our study draws on a crucial dive into the CC-process, exploring its core components (Theme 1) as represented within the triangle. Facilitating factors (Theme 2) are represented at the core of the framework.

The first essential component of the CC-process with diverse faculty is the participants’ immersion in positive feelings of inclusivity and appreciation. Although undergraduate curricula design is the sole responsibility of the college [28], GME stakeholders appreciated being involved in shaping the curricula of their future trainees. Connecting with UME stakeholders and students in transition was a great chance to inform them about postgraduate requirements. Workplace partners benefit from having a say in student preparation [9]. Additionally, they valued the input of UME to inform them about the students’ preparedness. Similarly, students appreciated their inclusion in the curriculum design, which extends previous research that revealed the effect of CC in enhancing learner-teacher relationships with an increased students’ sense of belongingness and cohesion [29], students’ self-authorship [30] and ownership [31]; our participants felt empowered and responsible for their peers as they spoke on their behalf. Finally, students expressed a feeling of relief and reduced stress as the CC exercise allowed them to identify their weaknesses and how to improve. This is in line with a previous study that showed an environment that empowers learners to share their needs, understands their expectations, and engenders feelings of respect, where emotions can be safely addressed, leading to positive well-being [2]. This promising finding adds to the value of CC, particularly within the transition context, perhaps an asset to reduce trainees’ stress during the changes entailed in transition [32–35].

Secondly, the sharing of knowledge in CC is highly valued as a learning experience and a crucial core component. It allows individuals with diverse experiences and levels of expertise to come together, which is essential in the context of transitions. The communication of knowledge was shown in previous work to be worthy of student handover aimed at closing the transition gap [14]. The study participants perceived the CC inclusion approach as fostering common ground and a shared-mental model, with shared understanding resulting in better curriculum quality. This was facilitated through the other notable third core component, the stimulation of self-reflection among all participants. Diverse faculty benefits students by helping them understand GME stakeholder expectations, reflect on weaknesses and gaps in their preparation, and identify ways to address those gaps. The pedagogical partnership between students and staff during CC is conceptualized “as a way to reflect and grow together, offering each other feedback and solving the puzzles of the class as a team” [36]. When given the opportunity to reflect on their experiences, students from across contexts become insightful and eloquent, and both develop respecting voices [37]. Furthermore, UME and GME stakeholders exchanged knowledge and expertise, which enabled them to reflect on their practices. Thus, moving from a two-way to a three-way conversation, as occurred during the CC-process in the presence of diverse faculty, may have challenged the deep and longstanding beliefs of both types of faculty, thus stimulating their self-reflection process for developing ideas to improve education [6, 38].

Our study emphasized the value of translation of CC dialogues into the intended outcomes as the fourth core component. Participants stated that failure to implement CC recommendations may result in a reluctance to participate again, raising the phenomenon of ‘voice fatigue’. This has been reported as learners’ reluctance to be involved in co-creation because involvement is accompanied by a high workload and time constraints [39]. Conversely, studies showed that learners dislike situations where their influence appears to be limited to giving advice without actual involvement in the implementation process [40].

It has been described that deeply rooted positions of faculty and students challenge power relations in CC [6, 41]. The presence of diverse faculty could worsen the situation as each stakeholder may take a position as emphasized by the Positioning Theory [18]. This could give rise to power relations driven by dual positions such as student-faculty and faculty-faculty. In our study, students experienced *initial* discomfort, because the diverse faculty members were future program directors and academic staff, which made them evaluators and experts in curriculum development. However, the UME did not perceive this, and GME responsible stakeholders were impressed with the student contribution with free and honest voices.

Our work identified several mitigating factors of dual power relations, embedded in the core of the modified framework (Fig. 1) to indicate its significance in shaping the CC-process. The most important mitigating factor is the respect between participants, which resonates with previous work that acknowledged that the expertise of both learners and teachers has to be valued [6, 42, 43] and both perspectives are equally appreciated in conversations [44]. Effective moderation was also highlighted by participants as an important factor in creating a positive relationship. This was previously flagged; sharing of power to negotiate the curriculum between students and staff was an important marker of success in staff-student collaborations [41, 45, 46]. Our study suggests moderators encourage openness, invite all to participate, and maintain respectful discussion, which is perceived as empowering and validating for participants. Finally, constructive feedback by teachers was also indicated as an important mitigating factor of the power dynamics. Studies showed that responding respectfully and appreciatively to learners’ input and feedback can frame CC into a mutual learning experience [47].

## Implications for practice

CC is employed to engage key stakeholders in shaping curricula. We urge school leaders to adopt an inclusivity approach and involve not only students and teachers but also workplace partners to provide the foundation for developing quality transition curricula. Achieving a shared vision requires acknowledging diverse perspectives. A strength of the present study is the unique inclusivity approach between UME and GME stakeholders with students in transition with a subsequent appreciation, sharing of knowledge, reflection, analysis, and translation of CC dialogues into the intended outcomes. Understanding the path that CC participants walk is crucial for educational leaders who seek to foster students’ voices through CC. Paying attention to the power dynamics and encouraging openness, respect, and constructive feedback through effective moderation is important to achieve the intended outcomes of CC. As such, this study offers a deeper insight into the process and the dynamics of the inclusion of multi-stakeholders and the measures that can reinforce the CC’s success.

## Limitations and future research

Certain limitations must be considered when interpreting our data. First, as with all qualitative research, the context of our study determines the findings: Different results may have evolved had we conducted it in a different context. Transferability is a concern, as the study findings can be culturally influenced. However, a thorough description of the context was made to enhance the transferability of the findings. Second, the recruitment of UME and GME stakeholders with final-year medical students provided richness to our findings. Had chief residents been available for interviews, we may however have gained different perspectives. Third, the findings were based on GME stakeholders, who are workplace partners. Whether the power dynamics will vary and be mitigated by the same factors if the CC setting includes other stakeholders such as nurses or patients merits investigation. It would be valuable to explore the effects of the four core components and the CC intended outcomes on the quality of curriculum design and experiences of all stakeholders after implementation. Finally, we encourage researchers to extend and explore the concept of CC in different transition periods across the professional training continuum.

## Conclusion

This study has broadened our comprehension of the co-creation process in the setting of diverse stakeholders. The process of co-creation is dynamic and includes essential components such as inclusivity and appreciation, knowledge sharing, reflection and analysis of current practices, and translation of co-creation dialogues into intended outcomes. It correspondingly embraces the presence of dual power dynamics and identifies measures to mitigate their effects such as the value of openness, respect, constructive feedback, and effective moderation. Engraining the co-creation process as the central part of the Framework of Stakeholder Involvement in Co-Creation is particularly important when diverse stakeholders are involved. The modified framework enables the utilization and enrichment of co-creation by inviting wider expertise and experience of all relevant stakeholders.

### Supplementary Information

Supplementary Material 1.

## Data Availability

The datasets generated and analyzed during the current study are available from the corresponding authors on reasonable request.
