# Supplementary material for: Achieving ‘something that everybody has invested in’: perspectives of diverse stakeholders during co-creation of a transition to residency curriculum
Source: BMC Med Educ. 2024 Jun 11;24:650. doi: 10.1186/s12909-024-05573-1 (PMC11167939; doi:10.1186/s12909-024-05573-1)
Supplement: Supplementary file 1 — Supplementary Material 1. [file 12909_2024_5573_MOESM1_ESM.docx]

**Appendix A. Faculty Interview guide**

Thank you so much for participating in the CC sessions. Please state which sessions you were involved in. The aim of this interview is to obtain your perception of the CC sessions experience. It is completely confidential, and recordings will be kept secure. No names will be identified in publications. Do you accept starting to record the session?


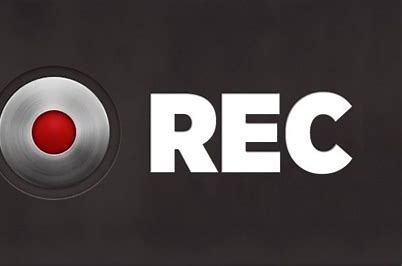


**Start recording**

**Question 1**

Have you been involved in CC sessions before? By CC, we mean: “ *“when staff and students work collaboratively with one another to create components of curricula and/or pedagogical approaches.”* What was your perception of these CC sessions with QU students? Please describe your experience. What was the quality of these sessions? What did you like most about it? What needs to be improved?

**Question 2**

From this experience, please describe your perception of the student’s contribution to the discussion.

- 1. How did you like the input of students? How did you feel about it? Were students’ contributions acceptable to you as a faculty? Did you feel offended by it? And why?
  2. Do you think students felt free to share their ideas and opinions?
  3. Do you think students felt responsible for contributing to the discussion?
  4. How did students’ contributions in the session affect the content of the discussions? Did students bring in points that you had not thought about? And in which way did students contribute to the co-created ideas for the curriculum?

**Question 3**

What was your perception of these CC sessions with the students and the College faculty/clinical faculty*? Please describe your experience. How did you perceive the inclusion of the college faculty/clinical faculty* in the CC session?

1. How did you feel about it?
2. Did you feel comfortable sharing the discussion and expressing your opinions? Please explain.
3. In which way do you think the college faculty/clinical faculty* had contributed to the discussion? Did they bring in points that you had not thought about? And what did they add to the co-created ideas for the curriculum?
4. Do you think Clinicians and College faculty gain any benefit if they sit together in CC sessions? Is it a learning opportunity for either/both of them? Explain why?

**Question 4**

How did you experience the session with only students compared to the session with students and college faculty/clinical faculty* (the last one)? Did you experience differences? What were the differences? Was it more/less difficult? More/less valuable?

**Question 5**

How did you perceive these CC sessions as being in an online format? What were the benefits and the challenges of using digital media as an environment for discussion?

1. Did you feel hindered to contribute to the discussion?
2. Did you feel any difference from face-to-face sessions?
3. How did you value the moderator? How did you find the quality of the facilitation? Did he or she allow the contribution of all participants? Did he/she make you feel comfortable expressing your opinions/ideas?
4. Were you satisfied with the quality of the net? Have you had any technical troubles?

**Question 6**

Having been through the experience of CC with medical students,

1. Would developing curricula/ courses/workshops or any other training using the CC concept in undergraduate education would be beneficial? Explain why?
2. Does CC have added benefits, especially during students’ transition (preclinical to clinical, clerkships to residency…etc.)? Explain why?
3. So, do you think it has an impact on reducing students’ stress and anxiety if they co-create the curriculum during the transition? Explain why?
4. Do you think this will add value to the training program at the College of Medicine? Would it make any difference in the quality of the training provided to the students?
5. Would you think this can be applied to postgraduate training, i.e., involving residents and fellows in the CC of the curriculum with program leaders and faculty?

*If you are interviewing clinical faculty, ask about your experience with college faculty and vice versa.

**Appendix B. Students Interview Guide**

Thank you so much for participating in the CC sessions. Please state which sessions you were involved in. This interview aims to obtain your perception of the CC sessions experience. It is completely confidential, and recordings will be kept secure. No names will be identified in publications. Do you accept starting the recording of the session?


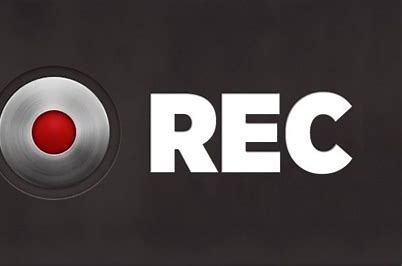


**Start recording**

Have you been involved in CC sessions before? By CC, we mean: “ *“when staff and students work collaboratively with one another to create components of curricula and/or pedagogical approaches.”* Have you been involved in CC sessions before? What was your perception of the QU CC sessions? Please describe your experience with these sessions.

1. What was the quality of these sessions?
2. Did you feel free/safe or comfortable speaking and contributing to the discussion? Why yes/no?
3. Did you feel your input was valued/listened to?
4. Did you feel your contribution added to the value of the curriculum? How is that?
5. Do you think there is any difference between a curriculum developed exclusively by college leaders and the one created by including the students? And how might they differ?
6. How did you perceive the CC session with the college staff and those with the clinical staff?
7. A Were there any differences in terms of your engagement? Did you feel more comfortable/ safe in the discussion in one than the other?
8. Did you feel any difference in the discussion or the generated ideas when comparing both?
9. Which session was most valuable and why?
10. How did you experience the session with both staff and clinicians together?
11. Did you experience differences in terms of your contribution? In terms of feeling safe/comfortable in the discussion?
12. Did you feel there is a difference in terms of generated ideas and the curriculum as a whole when the clinicians, university faculty, and students contribute together?
13. Which of the three sessions did you like most and why?
14. How did you perceive these CC sessions in an **online** format? What were the benefits and the challenges of using digital media as an environment for discussion?
    1. Did you feel hindered to contribute to the discussion? Why?
    2. Did you feel more or less safe to speak than in a face-to-face session? Why?
    3. How did you value the moderator? How did you find the quality of the facilitation? Did he or she allow the contribution of all participants? Did he/she make you feel comfortable expressing your opinions/ideas?
    4. Were you satisfied with the quality of the net? Have you had any technical troubles?
15. Having been through the experience of CC with medical students,
16. Would developing curricula/ courses/workshops or any other training using the CC concept in undergraduate education would be beneficial? Explain why?
17. Do you think this will add value to the training program at the College of Medicine? Would it make any difference in the quality of the training provided to the students? Explain why?
18. Would you think this can be applied to postgraduate training, i.e., involving residents and fellows in the CC of the curriculum with program leaders and faculty?
